# Hypoglycemic unawareness: challenges, triggers, and recommendations in patients with hypoglycemic unawareness: a case report

**DOI:** 10.1186/s13256-022-03498-1

**Published:** 2022-07-21

**Authors:** Yasaman Sharifi, Mahbube Ebrahimpur, Seyed Saeed Tamehrizadeh

**Affiliations:** 1 Endocrinology and Metabolism Research Center, Endocrinology and Metabolism Clinical Sciences Institute, Tehran University of Medical Sciences, First Floor, No 10, Jalal-Al-Ahmad Street, North Kargar Avenue, Tehran, 14117-13137 Iran; 2 Radiology Department, Iran University of Medical Sciences, Tehran, Iran; 3 Elderly Health Research Center, Endocrinology and Metabolism Population Sciences Institute, Tehran University of Medical Sciences, Tehran, Iran; 4 Tehran University of Medical Sciences, Tehran, Iran

**Keywords:** Diabetes, Hypoglycemic unawareness, Human insulin, Continuous glucose monitoring, Hypoglycemia-associated autonomic failure

## Abstract

**Background:**

Hypoglycemia is a fairly common complication in diabetic patients, particularly in those on insulin therapy. Hypoglycemia symptoms are classified into two types: autonomic and neuroglycopenic symptoms. If a person develops neuroglycopenic symptoms before the appearance of autonomic symptoms or is asymptomatic until blood sugar levels are very low, the patient will develop hypoglycemic unawareness (HU).

**Case presentation:**

A 25-year-old Iranian woman with HU presented with a severe hypoglycemic episode. This episode was characterized by loss of consciousness and focal neural deficits, which were unusual symptoms in the patient, who was a medical intern with type 1 diabetes and currently being treated with regular and NPH insulin.

**Conclusions:**

Hypoglycemia is a common complication in diabetic patients receiving oral or insulin therapy. A patient who is unaware of their condition may experience severe and potentially fatal episodes. These incidents can negatively affect their daily lives as well as their careers and jobs. Hypoglycemia-associated autonomic failure is a possible cause for patients with multiple episodes of severe hypoglycemia. IThe use of a continuous glucose monitoring device with an alarm, if available, can be an excellent option for these patients.

## Background

Hypoglycemia is a relatively common complication in diabetic patients, particularly those on insulin therapy [1]. Hypoglycemia symptoms are classified into autonomic and neuroglycopenic symptoms [2]. Autonomic symptoms are acetylcholine-mediated cholinergic symptoms, such as sweating, hunger, and paresthesia, or catecholamine-mediated adrenergic symptoms, such as palpitations, anxiety, systolic blood pressure change, and tremors at blood glucose (BG) levels of ≤ 70 mg/dl [1–3]. These autonomic symptoms serve as a warning signs [4]. Glucose is the primary fuel source of the brain. As BG levels decrease to ≤ 54 mg/dl, neuroglycopenic symptoms appear, although symptoms can also be seen at higher BG levels [5, 6]. These symptoms include a feeling of warmth (despite cold and damp skin), weakness, difficulty in thinking, tiredness and drowsiness, dizziness, blurred vision, slurred speech, loss of consciousness, as well as rare localized neurological conditions (diplopia and hemiparesis) [5]. Decreased or impaired awareness of hypoglycemia, commonly referred to as HU, is the development of neuroglycopenic symptoms without first having autonomic warning signs [6–8]. HU is more common in patients aged > 65 years who have a long history of diabetes (diabetes duration > 10 yers) [9]. Moreover, HU increases the risk of recurrent severe hypoglycemia by six- to ninefold in persons with type 1 or type 2 diabetics (T1DM, T2DM, respectively) [6, 7, 10].

Here, we present the case of a medical student with a history of T1DM being treated with human insulin who suffered severe neurological symptoms from HU.

## Case presentation

A 25-year-old Iranian woman (weight: 57 kg; body mass index: 20.54 kg/m^2^) was admitted to the hospital emergency room in Tehran, Iran after being discovered unconscious and unresponsive to sensory and pain stimulation. She was a medical intern at the same hospital in the internal medicine ward, and the episode happened at 3 a.m. at the end of her shifts, while she was sleeping in the doctors’ room. Because other colleges were unaware of her previous medical history, the protocol for unconscious patients was immediately implemented. Vital signs of the patient upon arrival in the emergency room were: blood pressure = 140/70 mmHg; heart rate = 110 bpm; oxygen saturation level = 98% on the right atrial; respiratory rate = 16 and temperature was 36.8 °C. The patient’s BG level was 25 mg/dl by self-monitoring blood glucose (SMBG) device. Since the patient was unconscious, she received an intravenous (IV) bolus of 50% dextrose. Laboratory tests were run, including thyroid, kidney and liver function tests; the results are shown in Table 1. All tests revealed no abnormalities, despite a low BG level. The insulin level was high with a low C-peptide level.

**Table 1:** Results of the blood examination on first admission

Variables	Value	Normal range
Random blood sugar (mg/dl)	25	60–100
Insulin (μIU/ml)	55.5	2.6–24.9
C-peptide (ng/ml)	0.3	1.1–4.4
TSH (μIU/mL)	0.7	0.3–5
FT4 (ng/dL)	0.98	0.67–1.5
Calcium (mg/dl)	9.1	8.6–10.3
Creatinine (mg/dl)	0.89	For adult women, 0.59–1.04
Blood urea nitrogen (BUN) (mg/dl)	13	6–24
SGOT (AST), U/l	26	7–56
SGPT (ALT), U/l	18	5–40

*TSH* Thyroid stimulating hormone, *FT4* thyroxine,* BUN *blood urea nitrogen, *SGOT (AST)* serum glutamic-oxaloacetic transaminase (aspartate aminotransferase), *SGPT (ALT)* serum glutamic pyruvic transaminase (alanine transaminase)

Uptake of the IV bolus of dextrose improved the patient's level of consciousness, but she still had focal neurological symptoms, including hemiparesis and aphasia. Fifteen minutes after the initiation of treatment with serum dextrose, she was responsive to pain and stimulation. She remained in the emergency room for monitoring, and after 1 h all neurological symptoms had disappeared, satisfying Whipple’s triad of hypoglycemia. Thus, a CT scan to rule out vascular events was deferred. After regaining consciousness, the patient mentioned having T1DM since the age of 18 years and receiving regular  treatment with insulin and Neutral Protamine Hagedorn insulin (NPH). In this setting, non-beta cell tumors are unlikely to be diagnosed. The patient also claimed experiencing HU for the previous 2 years. She also mentioned at least three episodes of severe hypoglycemic episodes weekly during the last 3 months that may have necessitated the assistance of others. These episodes mostly happened at night.

The patient claims that she was very active during her work shift and did not have time to eat adequately, but she injected insulin at the usual dose.

The injection regimen of the patient consisted of multiple insulin injections day: regular insulin, 10 U before breakfast and dinner, and 6 U before lunch; NPH insulin, 25 U in the morning and 10 U at night). Her dose had been adjusted at her last visit to her endocrinologist 3 months previously, but she has had several severe hypoglycemic attacks during the last 4 weeks. The patient’s lifestyle had also changed in the last 6 months, as she had begun routine gym exercise to manage her insulin needs and glycemic control.

She was examined by a neurologist in the morning for her focal neurological symptoms, and the examination revealed no deficits. Her medical history was also concerning for hpoglycemia-associated autonomic failure (HAAF), and she was recommended to have this condition evaluated as outpatient. To avoid recurrent hypoglycemia, further laboratory tests and a follow-up evaluation with an endocrinologist were recommended, as well a switch from human insulins to analog insulins.

## Discussion

Hypoglycemia is a common side effect of various diabetes medications, such as insulin and sulfonylureas [8, 11]. This condition can cause life-threatening episodes, significant morbidity, and a lack of optimal glycemic control. Many routine activities, such as driving, job performance, and sporting competitions, can be affected by hypoglycemia [12]. About 4% of patients with T1DM who use basal-bolus insulin regimens experience hospitalization for severe hypoglycemia. One study reported that the extra costs incurred by this patients per month amount to an average of US$712 (52%) after their hospitalization compared to outpatient patients or comparable patients [13].

Clinically, symptomatic hypoglycemia is diagnosed using Whipple’s triad, which includes symptoms of hypoglycemia, a plasma glucose level < 55 mg/dl and resolution of symptoms after the plasma glucose concentration has been raised [14]. Whipple’s has also been used to diagnose hypoglycemic attacks, and our patient met the Whipple criteria or hypoglycemia.

According to the American Diabetes Association (ADA), level 1 hypoglycemia is defined as a measurable glucose concentration < 70 mg/dl (3.9 mmol/l) but ≥ 54 mg/dl (3.0 mmol/l). In people without diabetes, a BG concentration of 70 mg/dl (3.9 mmol/l) has been recognized as the critical threshold for the initial appearance of autonomic symptoms [15]. Level 2 hypoglycemia, characterized as a BG concentration < 54 mg/dL (3.0 mmol/l), is the threshold at which neuroglycopenic symptoms occur, and immediate action is necessitated to resolve the hypoglycemic event. If a patient has level 2 hypoglycemia but no adrenergic or neuroglycopenic symptoms, he/she is most likely suffering from HU. This clinical scenario necessitates additional investigation and a review of the medical regimen. Level 3 hypoglycemia is defined as a severe event defined by altered mental and/or physical functioning that necessitates the assistance of another person to recover [15]. Recurrent level 2 and/or level 3 hypoglycemia is a medical emergency that requires intervention with medical regimen adjustments, behavioral interventions, and the use of diabetes technologies, including continuous glucose monitors (CGMs) and hybrid closed loops systems, which can also be utilized to help detect, predict and/or prevent hypoglycemia events [15–18]. According to these classifications, our presented case had level 3 hypoglycemia with unconsciousness, neurological symptoms, and a BG level of 25 mg/dl.

The true prevalence of hypoglycemia in persons with T1DM is unknown [2, 8, 19]. Studies indicate that severe hypoglycemia affects 35–42% of patients with T1DM, resulting in between 90 and 130 episodes for every 100 patients [10, 20, 21]. HU happens more often in those who: (1) repeatedly have low blood sugar episodes (which can cause the patient to stop sensing the early warning signs of hypoglycemia); (2) have had diabetes for an extended time; and (3) tightly control their diabetes (which intensifies their probabilities of having low blood sugar reactions) [15, 16, 18].

The causes of hypoglycemia in people with diabetes, include:

1. Changes to insulin regimen. (In our case, the patient did not mention the increase in the dose of insulin used and, according to the patient’s profession, the wrong amount and type of injectable insulin and accidental injection of insulin into the muscle is unlikely but possible) [1, 2, 6, 8, 11].
2. Decreased glucose that enters the bloodstream. (The possible explanation of the hypoglycemia in our patient is expected to be delayed meals due to work shifts and lack of carbohydrates at night before sleeping )[1, 2, 6, 8, 11].
3. Increased glucose uptake. (Other possible causes, in the present case, are due to increased physical activity following work shifts) [1, 2, 6, 8, 11].
4. Decreased endogenous glucose production following alcohol consumption. (The medical history of our patient and test results did not confirm this possibility) [1, 2, 6, 8, 11].
5. Decreased renal insulin excretion following renal failure. (The medical history of our patient and test results did not confirm renal insufficiency) [1, 2, 6, 8, 11].
6. Increased insulin sensitivity following weight loss or exercise or severe glycemic control. (Depending on the patient’s profession, there is a possibility of severe glycemic control due to recurrent hypoglycemia. She also mentioned beginning sports activities in the last 6 months) [1, 2, 6, 8, 11].

Another issue that needs to be investigated is the causes of hypoglycemia, which should be investigated based on the patient’s diabetes history. Previous studyies have linked both tight glycemic control [22–24] and attempts to rapidly control hemoglobin A1c (HbA1c) levels [22, 25] to increased hypoglycemic events [26]. Our patient had an HbA1c of 5.8%, which was lower than the appropriate diabetes control threshold in the patient’s age group and indicated a higher risk of hypoglycemia according to additional tests performed on the patient. According to related studies in patients with insulin-dependent diabetes, the incidence of hypoglycemic attacks in patients taking regular insulin is higher than that  in patients taking newer insulins, including lispro [27–29], which is consistent with our reported case. Our patient had also been given regular insulin and NPH. The risk of hypoglycemia is higher with human insulin than with analog insulin such as Lantus and Novorapid [30], and therefore the preferred type of insulin in T1DM is analog insulin. A study by Smith *et al.* revealed that reduced compliance to changes in insulin regimen in hypoglycemia unawareness is consistent with hypoglycemic stress habituation. These authors concluded that therapies aimed at altering repetitive risky behavior could be beneficial in restoring hypoglycemia awareness and preserving toward severe hypoglycemia [31].

HAAF is another possible explanation for the hypoglycemic episodes experience by our patient. HAAF is a type of functional sympathoadrenal failure caused most commonly by recent antecedent iatrogenic hypoglycemia and is at least partially reversible by careful avoidance of hypoglycemia. HAAF can be maintained by recurrent iatrogenic hypoglycemia [32]. A ≥ 25-fold increase in the risk of severe hypoglycemia is associated with the onset of HAAF during intensive glycemic therapy. It is vital to distinguish HAAF from conventional autonomic neuropathy, which can also be caused by diabetes. Sympathoadrenal activation appears to be inhibited only in response to hypoglycemia, while autonomic activities in organs, such as the heart, gastrointestinal tract, and bladder, are unaffected [32]. Our case was examined for this possibility due to her long history of severe hypoglycemic attacks, which needed further evaluation to rule out having HAAF after an evaluation of sympathoadrenal response to hypoglycemia.

People with HU are unable to detect drops in their blood sugar level, so they are unaware that they require treatment. Unawareness of hypoglycemia increases the risk of severe low blood sugar reactions (when they need someone to help them recover). People who are unaware of their hypoglycemia are also less likely to be awakened from sleep when hypoglycemia occurs at night. People who are hypoglycemic but are unaware of it must take extra precautions to monitor their blood sugar levels regularly. This is especially true before and during critical tasks, such as driving. When blood sugar levels are low or begin to fall, a CGM can sound an alarm. Such a device can be a great assistance to people with HU [12, 15]. With continuous BG monitoring, children and adults with T1DM spend less time in hypoglycemia and simultaneously decrease their HbA1c level [33, 34]. A prior study showed that diabetic patients with reduced beta-adrenergic sensitivity may be unaware of hypoglycemia, and the best suggestion for these patients is to strictly avoid hypoglycemia [35, 36]. Our patient was also advised to have emergency glucose tablets, intermuscular, or intranasal glucagon injections at her disposal all of the time to avoid hypoglycemic attacks. The glucagon injection pen was not available in Iran at the time of the episode described here, neither was a CGM, so she was recommended to follow educational sessions on carbohydrate counting and perform excessive SBGM.

The patient was given strict advice based on her job and profession, as well as the need to control her blood sugar level to the extent that it did not interfere with her professional and daily functioning [12]. She was advised to see her endocrinologist to adjust her insulin dose based on her unawareness of hypoglycemia attacks and her work schedule, which may not allow her enough time to rest and consume enough carbohydrates, potentially leading to life-threatening attacks, especially since her coworkers were unaware of her medical condition. It is strongly advised that people with diabetes, especially patients like this case, wear some sort of identification, such as a bracelet, or carry a card that state their condition [15].

Due to our patient’s extreme unawareness of the hypoglycemic attack, and given that one of the causes is the tight control of blood sugar and low numbers, the recommendation was that blood sugar numbers be raised to higher levels under the supervision of an endocrinologist for the regulation of the body’s natural reflexes. Normalization of autonomic response takes 7–14 days on average, but it can take up to 3 months to normalize the threshold of symptoms, neuroendocrine response, and glucagon response (although glucagon response is never fully recovered) [37, 38]. Another suggestion was to switch human insulin to the analog type of insulin. Implementing CGM if it is possible due to costs and this patient's career could be a valuable effort given the burden of HU in this patient and in others in every country’s healthcare system.

## Conclusions and learning points

Hypoglycemia is a fairly common complication in diabetic patients receiving oral or insulin therapy. However, in a subset of patients who are unaware of hypoglycemia for a variety of reasons, these warning signs do not exist, resulting in severe and life-threatening hypoglycemic episodes. These incidents negtivel affect people’s daily lives as well as their careers and jobs. Also, if other people are unaware of the person’s previous illness, there is a risk that they will act inappropriately if the patient’s level of consciousness declines. As a result, patients who have multiple episodes of HU are advised to raise their blood sugar control threshold for at least 2 weeks and to wear at all times a bracelet or label indicating their medical condition. In addition, in these patients, the use of CGM equipped with alarms in the occurrence of severely low blood sugar can be a perfect option.

## Data Availability

Patient data and information can be accessed for review after obtaining permission from the patient without any disclosure of her name.
